# PHB production from cellobiose with *Saccharomyces cerevisiae*

**DOI:** 10.1186/s12934-022-01845-x

**Published:** 2022-06-21

**Authors:** Anna Ylinen, Jorg C. de Ruijter, Paula Jouhten, Merja Penttilä

**Affiliations:** 1 VTT Technical Research Centre of Finland Ltd., P.O. Box 1000, 02044 Espoo, Finland; 2 Present Address: Department of Bioproducts and Biosystems, School of Chemical Engineering, Aalto University, P.O. Box 16100, 00076 Espoo, Finland; 3 Department of Bioproducts and Biosystems, School of Chemical Engineering, Aalto University, P.O. Box 16100, 00076 Espoo, Finland

**Keywords:** Polyhydroxybutyrate, *Saccharomyces cerevisiae*, Cellobiose, Cellobiose phosphorylase, β-glucosidase

## Abstract

Replacement of petrochemical-based materials with microbially produced biodegradable alternatives calls for industrially attractive fermentation processes. Lignocellulosic materials offer non-edible alternatives for cultivated sugars, but require often use of expensive sugar releasing enzymes, such as β-glucosidases. These cellulose treatment costs could be reduced if microbial production hosts could use short cellodextrins such as cellobiose directly as their substrates. In this study, we demonstrate production of poly(hydroxybutyrate) (PHB) in yeast *Saccharomyces cerevisiae* using cellobiose as a sole carbon source. Yeast strains expressing PHB pathway genes from *Cupriavidus necator* and cellodextrin transporter gene *CDT-1* from *Neurospora crassa* were complemented either with β-glucosidase gene *GH1-1* from *N. crassa* or with cellobiose phosphorylase gene *cbp* from *Ruminococcus flavefaciens*. These cellobiose utilization routes either with Gh1-1 or Cbp enzymes differ in energetics and dynamics. However, both routes enabled higher PHB production per consumed sugar and higher PHB accumulation % of cell dry weight (CDW) than use of glucose as a carbon source. As expected, the strains with Gh1-1 consumed cellobiose faster than the strains with Cbp, both in flask and bioreactor batch cultures. In shake flasks, higher final PHB accumulation % of CDW was reached with Cbp route (10.0 ± 0.3%) than with Gh1-1 route (8.1 ± 0.2%). However, a higher PHB accumulation was achieved in better aerated and pH-controlled bioreactors, in comparison to shake flasks, and the relative performance of strains switched. In bioreactors, notable PHB accumulation levels per CDW of 13.4 ± 0.9% and 18.5 ± 3.9% were achieved with Cbp and Gh1-1 routes, respectively. The average molecular weights of accumulated PHB were similar using both routes; approximately 500 kDa and 450 kDa for strains expressing either *cbp* or *GH1-1* genes, respectively. The formation of PHB with high molecular weights, combined with efficient cellobiose conversion, demonstrates a highly potential solution for improving attractiveness of sustainable polymer production using microbial cells.

**Supplementary Information:**

The online version contains supplementary material available at 10.1186/s12934-022-01845-x.

## Introduction

Microbially made poly(hydroxyalkanoates) (PHAs) are a group of fully biobased and biodegradable polymers with versatile properties fitting different applications, ranging from medical sutures and scaffolds to single use packing materials and durable consumables. One of the most common PHAs is poly(hydroxybutyrate) (PHB), with properties similar to polypropylene. It consists of 3-hydroxybutyryl monomers, which are combined in living cells to long linear polymer chains, reaching up to 10–20 thousand monomers per molecule. Polymerization starts from merging two acetyl-CoA molecules into one acetoacetyl-CoA by an acetyltransferase (PhaA). Acetoacetyl-CoA is then further converted to 3-hydroxybutyryl-CoA by an acetoacetyl-CoA reductase (PhaB1) and added covalently to the growing polymer chain by a PHA synthase (PhaC1). The synthesis of PHB has been shown in native and engineered bacteria and yeast species [1–3]. Optimization of cultivation conditions and engineering of cofactor supply and the cell wall flexibility have increased PHB accumulation in bacterial hosts up to 90–94% PHB per cell dry weight (CDW) [3–5]. Development of yeast strains as hosts for PHA production is a more recent approach. However, robust yeasts have many advantages for industrial use, e.g., insusceptibility for phase contamination, tolerance for acidic conditions, and ability to grow on inexpensive media [6–8]. We previously engineered a yeast *Saccharomyces cerevisiae* for PHB synthesis and were able to achieve up to 11% PHB accumulation of CDW from glucose [9].

*Saccharomyces cerevisiae* has the innate capability to efficiently ferment simple sugars, like glucose and sucrose, often deriving from edible crops like corn or sugarcane [10]. However, for economic and sustainability reasons, progress has been made to engineer this yeast to utilize carbon from non-edible crops [11]. The main components of more complex cellulosic feedstocks are cellulose, hemicellulose, and lignin. The release of glucose from cellulose requires large quantities of cellulase enzyme cocktails, including expensive β-glucosidase, which reduce the cost-effectiveness of the process [12]. However, Galazka and coworkers have shown that a recombinant expression of a cellodextrin transporter gene *CDT-1* from *Neurospora crassa* and an intracellular β-glucosidase gene *GH1-1* from the same organism allows *S. cerevisiae* to directly ferment cellodextrins, including cellobiose [13].

**Fig. 1:**
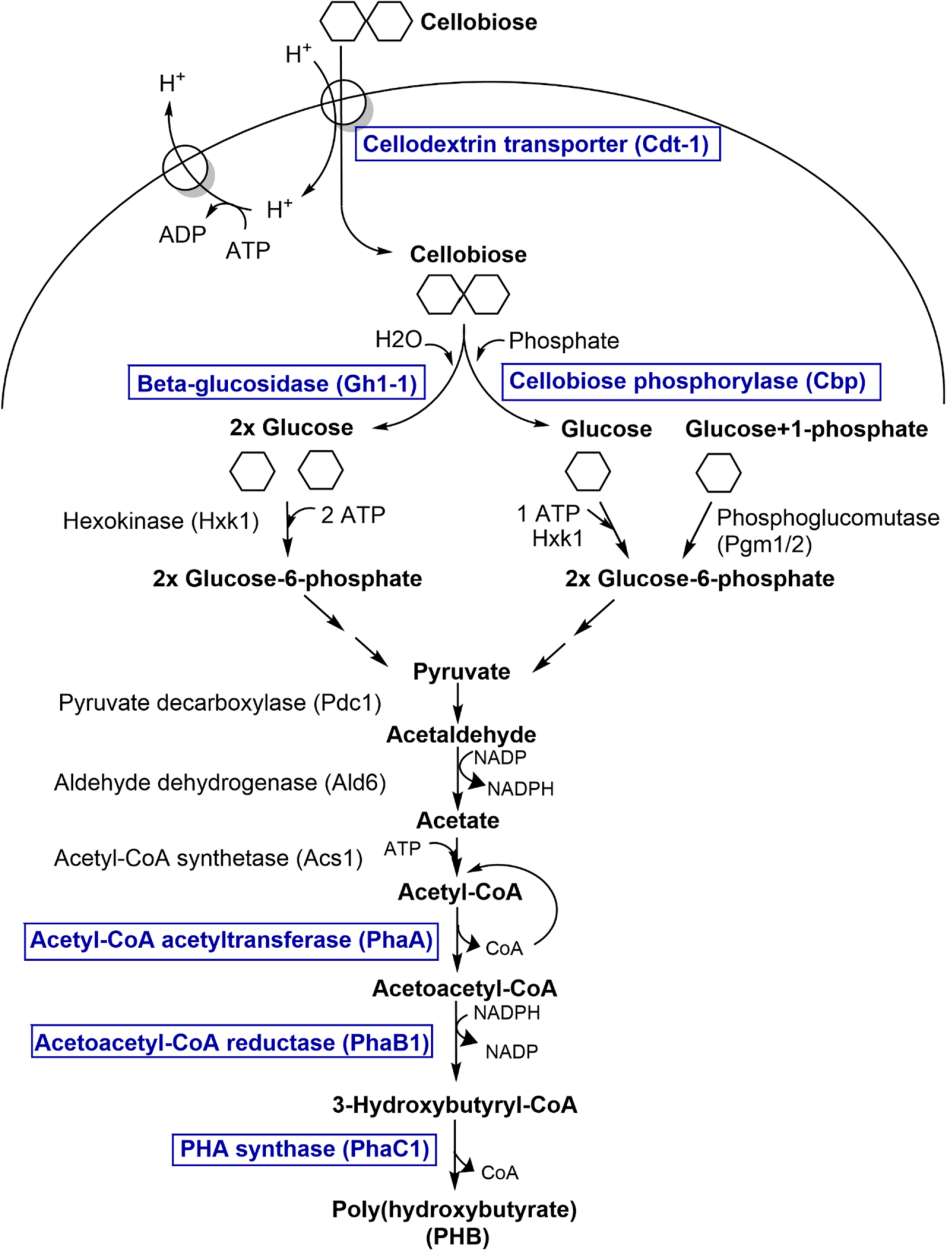
Engineered pathway for cellobiose conversion to polyhydroxybutyrate (PHB) in *Saccharomyces cerevisiae*. Genetic modifications are highlighted in boxes

The cellobiose molecules are cleaved into two glucose molecules by Gh1-1 and then phosphorylated by hexokinase Hxk1 consuming two ATP molecules, to enter into the glycolysis pathway (Fig. 1, left) [14]. The cellobiose pathway has been shown to be more energy efficient when the β-glucosidase is replaced by a cellobiose phosphorylase (Cbp), albeit with slower fermentation kinetics [15]. In this case, the cellobiose is phosphorolytically cleaved into glucose-1-phosphate and glucose. Therefore, with cbp, only one ATP is needed to generate two glucose-6-phosphate molecules for use in glycolysis (Fig. 1, right). Substantial improvement of the cellobiose fermentation kinetics with cbp was demonstrated using an evolved version of the *N. crassa* Cdt-2 cellobiose facilitator [16]. Cellobiose fermentation by *S. cerevisiae* has been further improved for example by screening for novel transporters and glucosidases [17, 18], or employing random mutagenesis and adaptive laboratory evolution [19, 20]. Ethanol titers of over 30 g l^−1^ [15, 16, 19, 21] and a productivity of over 1 g l^−1^ h^−1^ [19] have been reported, which demonstrates cellobiose as a suitable substrate for the production of chemicals using *S. cerevisiae*. Indeed, cellobiose utilizing *S. cerevisiae* strains have also been shown to efficiently produce 2,3-butanediol [22] and lactic acid [23].

In this study we demonstrate the production of biopolymer PHB by *S. cerevisiae* using cellobiose as a sole carbon source. We show the PHB production with two intracellular cellobiose utilization pathways, employing either a cellobiose phosphorylase or a β-glucosidase.

## Materials and methods

### Strains and media

Yeast strains, plasmids, and genes used are listed in Table 1. PHB production was shown in our previous study with *S. cerevisiae* strain CEN.PK113-5D carrying a PHB pathway in 2 µ plasmid with *URA3* selection [9]. Due to variation between transformation isolates and reported population heterogeneity in other plasmid strains [24, 25], we chose to produce PHB with a strain carrying the PHB pathway integrated to its genome. PHB pathway consisting of *phaA*, *phaB1*, and *phaC1* genes, was cloned into EasyClone expression vector pCfB3034 [26] using Gibson Assembly kit (E2611S, New England BioLabs). All plasmids were constructed in TOP10 *E. coli* cells by growing cells overnight in Luria–Bertani medium containing 100 mg l^−1^ ampicillin. *S. cerevisiae* CEN.PK parent strains were kindly provided by Dr. P. Kötter (Institut für Mikrobiologie, J.W. Goethe Universität Frankfurt, Germany). EasyClone expression plasmids were linearized with NotI enzyme (FD0596, Thermo Scientific) and transformed into yeast cells using CRISPR/Cas9 protocol of the EasyClone kit [26] and lithium acetate method [27]. Correct integration was confirmed with the EasyClone kit primers. The *N. crassa GH1-1* gene were integrated into strains PHB_GH1-1 and GH1-1_control as described previously [28]. The *R. flavefaciens* gene *cbp* was inserted into a MoClo expression vector, containing a *PGK1* promoter, *ENO1* terminator, 2-micron replication, and *LEU2* selection [29]. Primers oJR025 and oJR026 were used for creating an integration cassette with *URA3* flanks. Correct integration was confirmed with the primer pairs oJR004 and oJR064, and oJR064 and oJR065 (Table 1). All genes were codon optimized for expression in *S. cerevisiae*.

**Table 1:** Yeast strains, plasmids, and genes used in this study

Strains			
Name	Code	Description	References
Parent strains			
CEN.PK111-9A	H3892	*S. cerevisiae* (*MATa his3-Δ1 URA3 leu2-3,112 TRP1 MAL2-8c SUC2*)	^a^
CEN.PK113-7D	H3887	*S. cerevisiae* (*MATa HIS3 URA3 LEU2TRP1 MAL2-8c SUC2*)	^a^
PHB production strains			
PHB_glu	H5696	H3887 with integration of *phaA, phaB1*, and *phaC1* genes into X-3 EasyClone locus (plasmid B11787)	This article
PHB_cbp	H5716	H3892 with integration of *phaA, phaB1*, and *phaC1* genes into X-3 EasyClone locus and *ura3Δ*::*cbp* (from plasmid B9403), *CDT-1* (plasmid B8444)	This article
PHB_GH1-1	H5717	H3892 with integration of *phaA, phaB1*, and *phaC1* genes into X-3 EasyClone locus, *ura3Δ::GH1-1* (from plasmid pSS20), *CDT-1* (plasmid B8444)	This article

Control strains			
cbp_control	H5718	H3892 *ura3Δ*::*cbp* (from plasmid B9403), *CDT-1* (plasmid B8444)	This article
GH1-1_control	H5719	H3892 *ura3Δ::GH1-1* (from plasmid pSS20), *CDT-1* (plasmid B8444)	This article

Plasmids		
Code	Description	References
B11787	*pTEF1-phaA-tENO1-pTDH3-phaB1-tSSA1-pPGK1-phaC1-tCYC*	This article
B9403	*pPGK1-cbp-1-tENO1*, *LEU2,* 2µ, *kanR*	This article
pSS20	*pADH1-GH1-1-tENO1*, *LEU2, CEN/ARS, kanR*	[28]
B8444	*pPGK1-CDT-1-tENO1*, *URA3, CEN/ARS, kanR*	[28]

Primers		
Code	Sequence	References
oJR004	CCGACATAAGCTGGACCAGTA	This article
oJR025	TTGCCCAGTATTCTTAACCCAACTGCACAGAACAAAAACCTGCAGGAAACGAAGATAAATCGGAAAGAGTGAGGAACTATCGCATA	This article
oJR026	TAATTAAATTGAAGCTCTAATTTGTGAGTTTAGTATACATGCATTTACTTATAATACAGTTTTTCTGCCTATTTAACGCCAACGTTG	This article
oJR064	ACGAAGGAAGGAGCACAGAC	This article
oJR065	GACCGAGATTCCCGGGTAAT	This article

Genes	Code	Source	References
Name			
Acetyl-CoA acetyltransferase	*phaA*	*Cupriavidus necator*, GenBank KP681582	[30]
Acetoacetyl-CoA reductase	*phaB1*	*C. necator,* GenBank KP681583	[30]
PHA synthase	*phaC1*	*C. necator*, GenBank KP681584	[30]
Cellobiose phosphorylase	*cbp*	*Ruminococcus flavefaciens* FD-1, GenBank NZ_ACOK01000116.1	[31]
β-glucosidase	*GH1-1*	*Neurospora crassa,* NCU00130, GenBank 3872338	[13]
Cellobiose transporter	*CDT-1*	*N. crassa,* NCU00801, GenBank 3879950	[13]

^a^Strains were kindly provided by Dr. P. Kötter (Institut für Mikrobiologie, J.W. Goethe Universität Frankfurt, Germany)

### Shake flask cultivations

All strains were grown on synthetic complete media (SC, 6.7 g l^−1^ yeast nitrogen base), supplemented with amino acid mixture lacking uracil (SC-URA). Culture media were supplemented either with 20 g l^−1^ glucose or 20 g l^−1^ cellobiose, and cultivations were carried out at 30 °C with 220 rpm shaking. In the first flask experiment, precultures on cellobiose were grown for around 60 h, followed by a ten-fold dilution and second preculture on cellobiose of 24 h. This second preculture step was removed in later experiments.

For the EnPump 200 (Enpresso GmbH) slow glucose release experiments, 20 g l^−1^ EnPump substrate was dissolved into the synthetic complete media, followed by sterilization through microfiltration. After inoculation of the cultures slow release of glucose was started through the addition of Reagent A to a final concentration of 0.5 U l^−1^.

In most of the experiments, three parallel replicates were analyzed simultaneously, only exception being the first flask experiment, where only two replicates of strain PHB_glu were grown. Cell growth and metabolite production were analyzed once or twice a day from 1 ml sample. In the end of the cultivation, collected cells were washed with water and lyophilized overnight.

### Bioreactor fermentations

Yeast precultures were grown in 250 ml flasks in SC-URA media containing 20 g l^−1^ cellobiose. The cells were washed with water before resuspension in 50 ml of sterile deionized water for inoculation in the bioreactors at OD_600_ = 1.0. The batch cultivations were performed in 1 l Sartorius Biostat-Q benchtop bioreactors, with a final working volume of 750 ml. The strains were grown in SC-URA media with 35 g l^−1^ cellobiose. Temperature was set at 30 °C. Aeration was maintained at flow rate 1.5 l min^−1^ filtered (0.2 µm) ambient air, with the impeller rotation set to 600 rpm and the pH was maintained at 6 using either 1 M NaOH or 10% phosphoric acid.

### Analysis of cell growth, sugar consumption, and formation of metabolites

Cell growth was measured either as optical density (OD) at 600 nm with VitroSpec 2100 Pro (Amersham Biosciences) or by measuring cell dry weight (CDW). To obtain CDW, glass microfiber filter papers (55 mm, GF/B Whatman) were dried at 100 °C and weighted prior to filtration of 2–8 ml of sample. Filters with samples were dried over night at 100 °C and weighted. The CDW values of the flask cultivations were predicted with linear regression model from the measured OD_600_ and CDW values in the bioreactor experiment (Additional file 1: Fig. S1). As data points from 0 to 168 h from the strains PHB_cbp, cbp_control, PHB_GH1-1, and GH1-1_control aligned well to the linear model, the R-squared being approximately 0.966, the formula CDW (g l^−1^) = 0.3181* OD_600_ − 0.0463 was used for predicting the CDW values of the flask cultivations.

The pH results from flask cultivations were obtained with Innolab pH instrument (720, WTW) and Sentix Mic electrode (WTW). Metabolite production and components of the cultivation media (ethanol, acetate, glycerol, and glucose) were measured with high-performance liquid chromatography (HPLC). Samples were centrifuged at 13,500 rpm and supernatants were dissolved into 50 mM sulfuric acid in 1:1 ratio. The 5 mM sulfuric acid eluent was run with 0.5 ml min^−1^ flow rate at 55 °C. Metabolites were separated with Fast Acid Analysis Column (100 × 7.8 mm, BioRad Laboratories), an organic acid analysis column (300 × 7.8 mm, Aminex HPX-87H, BioRad Laboratories), and a separation module (2690, Waters). Peaks were detected with a differential refractometer (2414, Waters). Waters Empower 3 software was used for data processing. The measured cellobiose concentration in PHB_GH1-1 s bioreactor replicate at 72 h (27.1 g l^−1^) was considered as outlier due the technical measurement error and excluded from the data set. However, as the 72-h value was important for evaluation the biomass and PHB yield formation during the fast growth phase, it was estimated based on earlier and later results from the same bioreactor. Those results aligned well (R^2^ = 0.999) to polynomial function cellobiose (g l^−1^) = 0.0000223 * h^3^ − 0.0053783 * h^2^ + 34.4676 (Additional file 1: Fig. S2). Similar function was calculated from the results of the other bioreactor replicate and all the measured values aligned well to the polynomial function.

Cellotriose and cellotetraose measurements were done with a Dionex ICS-6000 High Pressure Ion Chromatograph (HPIC) system (Thermo Scientific). Samples were centrifuged at 13,500 rpm and supernatants were diluted 100 or 500 times in water. Samples were separated using a Dionex CarboPac Sa10-4 µm column at 40 °C with a 0.380 ml min^−1^ flow rate and 12 mM KOH for 15 min.

In bioreactor experiment, the measured extracellular cellotriose and cellotetraose values were subtracted from the measured total cellobiose consumption to calculate amount of cellobiose, which was consumed by the cells e.g., for growth and PHB production. These cellular cellobiose consumption values were used for evaluation of biomass and PHB yields per consumed cellobiose.

Determination of growth rates, specific growth rates, and specific production rates was done computationally using Matlab (Matlab 2019b, The Mathworks Inc.) and the Curve Fitting Toolbox. The measurement time series were smoothed and interpolated through cubic splines, and all rates and maximum values subsequently calculated from these smoothed curves. The different behavior of compared strains was confirmed with two-tailed students test using t.test function for similar variances (Excel 2016, Microsoft).

### Polymer quantification with GC–MS

The produced PHB per cell dry weight was analyzed with a gas chromatography mass spectrometry (GC–MS) method [32]. Cells collected from cultivations were centrifuged at 4000 rpm for 6 min, supernatants were removed, and cells were washed two times with distilled water. Samples were lyophilized for 24–48 h and 10 mg of each sample was subjected to methanolysis by heating at 100 °C for 140 min in a solution containing 1 ml chloroform, 150 µl sulfuric acid, 20 µl internal standard (3-hydroxybutyric acid), and 830 µl methanol. In addition, 3-hydroxybutyric acid was treated similarly as a reference sample. After samples were cooled to room temperature, the water-soluble particles were removed with 0.5 ml of water. Gas chromatography system (7890, Agilent) and HP-FFAP column (19091F-102, Agilent) was used for analyzing the chloroform phase.

### Polymer extraction

PHB polymer was extracted from cells by boiling lyophilized cell samples of 600 mg with 6–10 ml chloroform in glass tubes in 95 °C water bath for three hours [33]. After boiling, solutions were mixed using PTFE magnetic stir bars for 12–18 h in room temperature and filtered with 0.45 µm PTFE filters. Filtrate was concentrated to 300 µl in glass tubes and 7 ml of ice-cold methanol was added to precipitate the polymer. Tubes were centrifuged for 20 min at 3000 rpm. After removal of supernatant remaining polymer sample was dried and weighted.

### Size exclusion chromatography

Size exclusion chromatography (SEC) was used for analyzing molecular weights. PHB samples were dissolved in chloroform and mixed at room temperature for 5 days before filtration with 0.45 µm syringe filters. The chloroform eluent was run at 40 °C at a rate of 0.5 ml min^−1^. The system contained a pre-column, columns (Styragel HR-4 and HR 3, Waters) and a refractive index detector (2414, Waters). Ten polystyrene standards with molecular weights from 1.26 to 3040 kDa (kg mol^−1^) (Agilent) were used. Results were obtained using 3rd order fit (R2 = 0.998–0.999) and Waters Empower 3 software.

## Results

### Flask cultivations

The PHB producing *S. cerevisiae* strain was constructed by integrating the PHB pathway from *C. necator* into the yeast genome (Fig. 1)*.* Cellobiose utilization was enabled by integrating either the β-glucosidase gene *GH1-1* from *N. crassa* or the cellobiose phosphorylase gene *cbp* from *R. flavefacien*s. In addition, the *N. crassa* cellodextrin transporter gene *CDT-1* was expressed from a low copy *CEN/ARS* plasmid, because having only a single integration copy of the transporter was shown to limit cellobiose consumption in *S. cerevisiae* strains [19, 34].

**Fig. 2:**
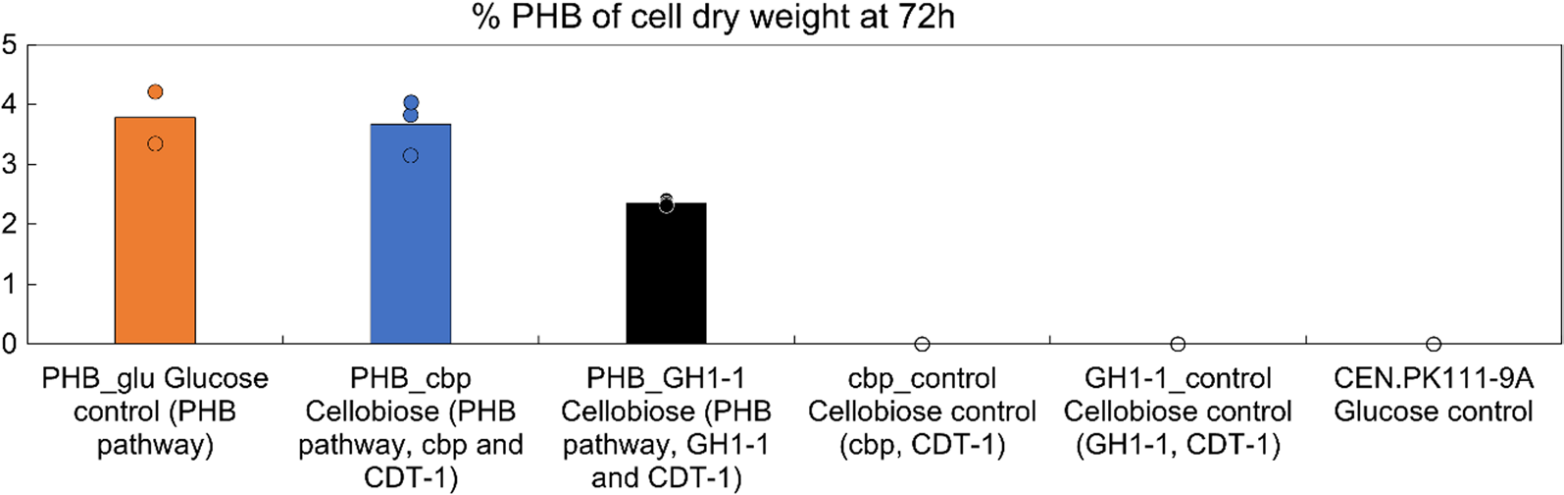
Polyhydroxybutyrate (PHB) yield % per cell dry weight (CDW) at 72 h. PHB producing strains and their corresponding control strains were grown in shake flasks either on 20 g l^−1^ glucose or on 20 g l^−1^ cellobiose

**Fig. 3:**
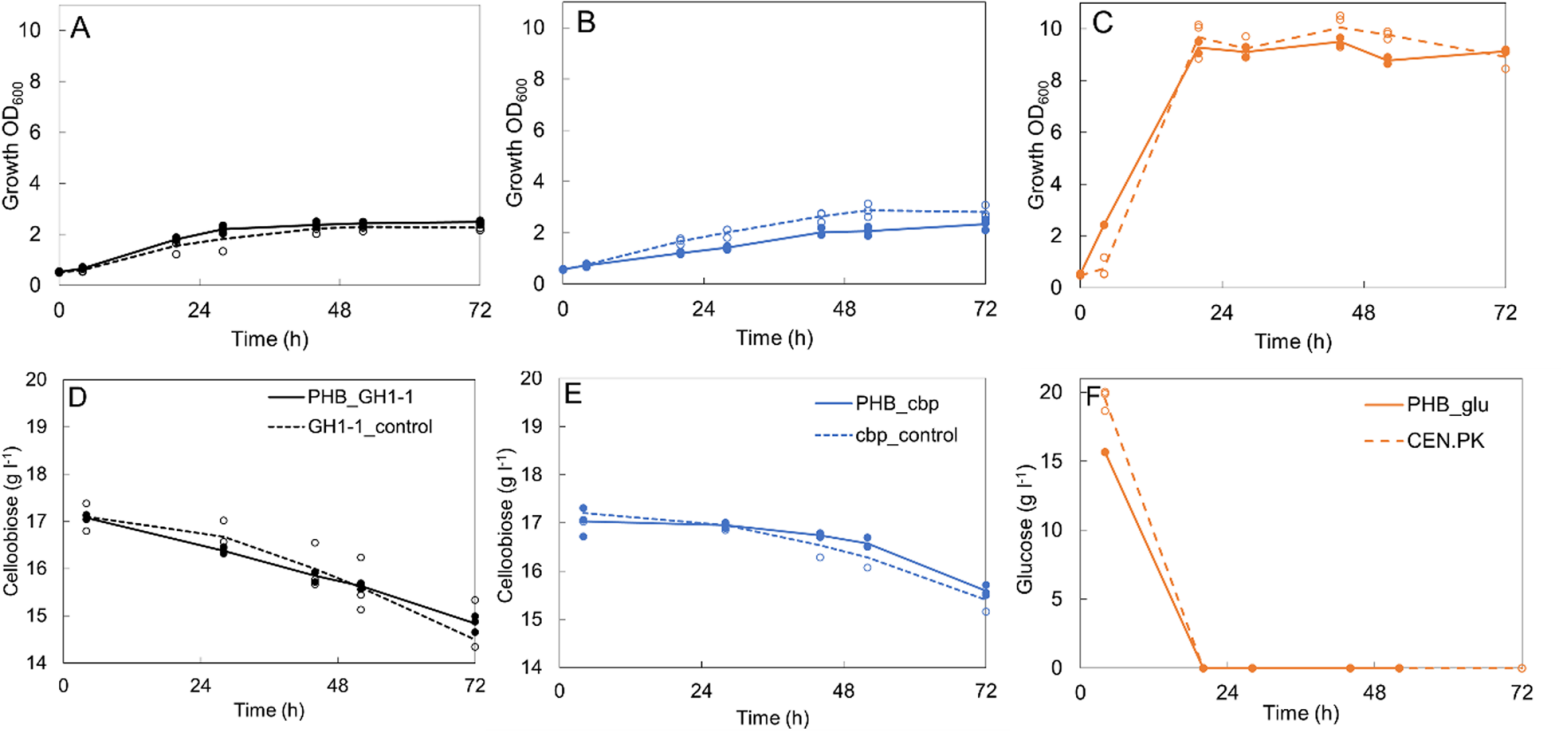
Cell growth and sugar consumption in shake flasks during the 72-h cultivation. Strains PHB_cbp and PHB_GH1-1 with their corresponding control strains were grown on synthetic complete (SC) media with 20 g l^−1^ cellobiose and strains PHB_glu and CEN.PK111-9A with on SC media with 20 g l^−1^ glucose. Individual data points are presented with circles to visualize the range of measured data

We compared cellobiose utilization, growth, and PHB production of strains with and without the PHB and cellobiose pathways in shake flask cultivations. The presence or absence of the PHB pathway did not notably affect the growth of the strains on cellobiose. Strains expressing the PHB synthesis pathway alone (strain PHB_glu) or with *CDT-1* transporter gene and either *cbp* (strain PHB_cbp) or *GH1-1* (strain PHB_GH1-1) gene produced 3.8, 3.7, and 2.4% PHB of cell dry weight (CDW) in three days, respectively (Fig. 2). As expected, PHB was not detected in control strains without the PHB pathway. In 72 h, the PHB_GH1 strain grew to OD_600_ 2.5 ± 0.1 and the PHB_cbp strain to OD_600_ 2.3 ± 0.2 (Fig. 3A, B). These strains consumed 2.6 and 1.4 g l^−1^ cellobiose, respectively, between 4 and 72 h (Fig. 3D and E). For comparison, the strain PHB_glu did not grow in the same media containing only cellobiose as a carbon source (Additional file 1: Fig. S5). However, when strains CEN.PK111-9A and PHB_glu were grown on 20 g l^−1^ glucose, all the carbon was consumed during the first 24 h of the cultivation (Fig. 3F). By 72 h, the biomass yield of cellobiose consuming strains per consumed cellobiose was 1.9–3.5 times higher than biomass yield of control strains per consumed glucose (p < 0.01) (Additional file 1: Table S1). However, when grown on cellobiose the strains reached approximately four-fold lower biomass concentration than on glucose (p < 0.0001). This lower final cell density on cellobiose reflected also to lower final PHB titer of around 20 mg l^−1^, in comparison strain PHB_glu on glucose (108 mg l^−1^). The possible cellotriose or cellotetraose production with the strains expressing *GH1-1 gene* was not measured in this experiment. However, we observed in a later bioreactor experiment that by 72 h, the cells expressing *GH1-1* gene had converted approximately 30% of the consumed cellobiose to longer cellodextrins. If such conversion occurred in the shake flasks, the PHB and biomass yields per consumed cellobiose would be higher than presented in Additional file 1: Table S1. However, this would not decrease the high 1.4 and 3.3-fold differences in biomass yield per consumed c-mol sugar on cellobiose (PHB_GH1-1, PHB_cbp strains, respectively) and on glucose (PHB_glu) (Additional file 1: Table S1).

**Fig. 4:**
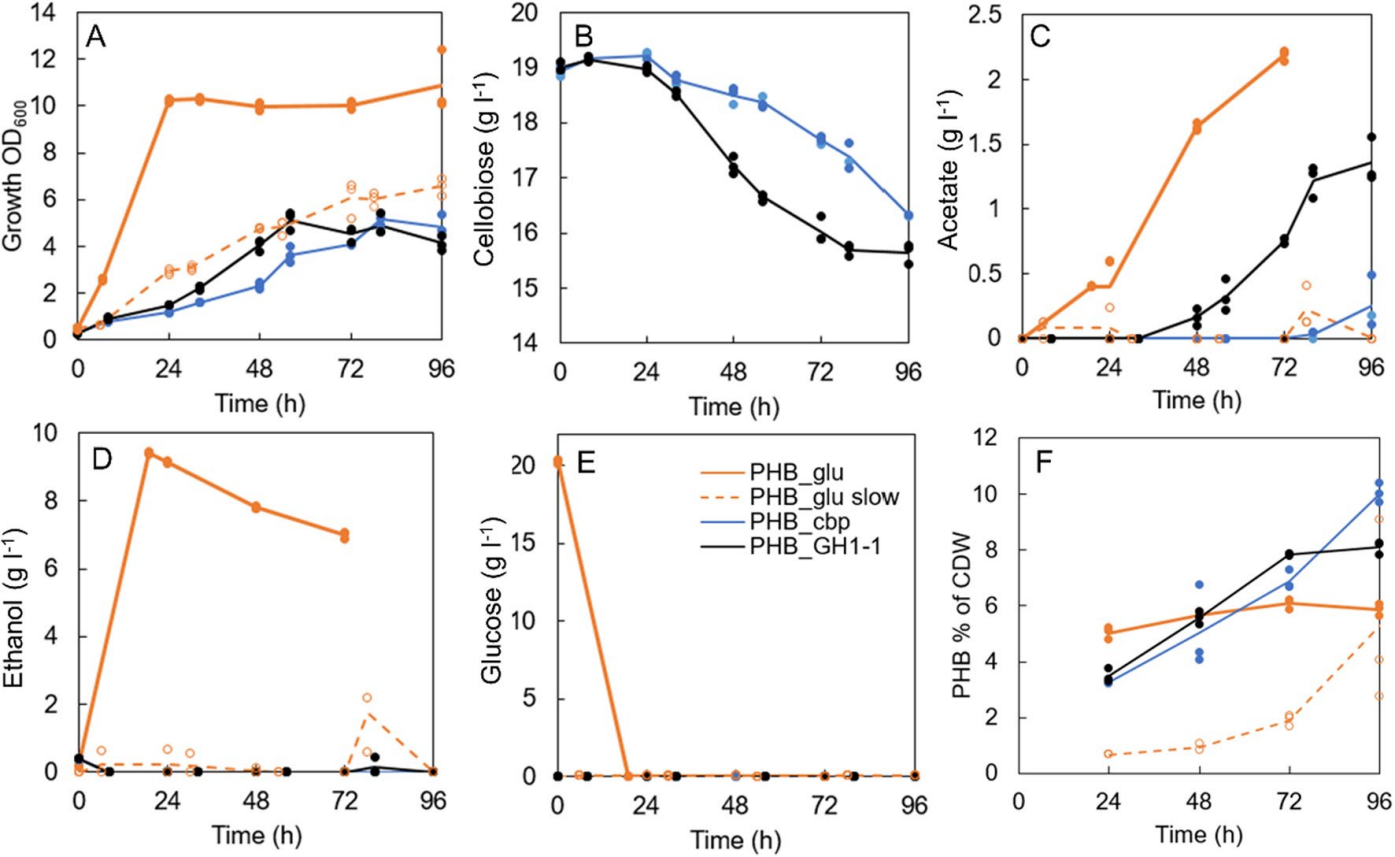
Results from 96 h shake flask cultivation. A: cell growth as OD_600,_ B: Cellobiose consumption (PHB_cbp and PHB_GH1-1 strains), C-D: Acetate and ethanol production, E: Glucose consumption (PHB_glu strain grown on 20 g l^−1^ glucose), F: PHB accumulation per cell dry weight (CDW). The PHB_cbp and PHB_GH1-1 strains were grown on 20 g l^−1^ cellobiose and PHB_glu strain either on 20 g l^−1^ glucose (PHB_glu) or with EnPump 200 system (PHB_glu slow). Lines represent averages of two or three biological replicates. Individual data points are presented with circles to visualize the range of measured data

Shake flask cultivation was repeated with the PHB producing strains to confirm PHB production on cellobiose and differences in PHB production on cellobiose and glucose. The strain PHB_GH1-1 consumed twice as much cellobiose, 2.9 g l^−1^, from 32 to 80 h, compared to PHB_cbp strain, 1.4 g l^−1^ (Fig. 4B) (p < 0.001). Strain PHB_GH1-1 also grew to higher OD (OD_600_ 5.13) during the first 56 h (p < 0.01), but by the 96 h, the strain PHB_cbp had reached similar cell density (OD_600_ 4.84) (Fig. 4A). However, ethanol production (9.36 g l^−1^) and fast growth (OD_600_ 10.2 by 24 h) were observed only when the cells were grown on glucose (Fig. 4A and D). The high difference in cellobiose and glucose consumption rates (Fig. 4B and E) inspired us to study PHB production also with an EnPump 200 system that provided glucose to the strains in similar rate to cellobiose (Additional file 1: Fig. S3). No glucose accumulation was observed with EnPump 200 media indicating that all released glucose was immediately consumed. The PHB_glu slow strain grown on EnPump 200 media produced less than 1 g l^−1^ of ethanol during the first three days. Around 0.6–2.4 g l^−1^ ethanol was produced after the third day, but all ethanol was consumed by the fourth day.

The PHB levels were measured every 24 h for four days (Fig. 4F). The PHB_cbp and PHB_GH1-1 strains grown on cellobiose accumulated PHB steadily during the entire cultivation and reached highest PHB levels of 10.0% and 8.1% of CDW, respectively, by the end of the cultivation. These accumulation levels were approximately 70% and 40% higher than in PHB_glu strain grown on 20 g l^−1^ glucose (6.1% of CDW) (p < 0.001), which produced most of the PHB already during the first day of the cultivation. The strain PHB_glu grown on EnPump 200 media accumulated least PHB, only 1.9% of CDW during the first three days. However, later, when the growth was ceased, PHB_glu with EnPump 200 media showed high variation in observed PHB accumulation: 2.8–9.7% PHB of CDW (Fig. 4F). The lowest PHB accumulation on EnPump 200 media resulted also in lowest PHB titers (mg l^−1^), even if cells grew slightly more than strains on cellobiose (Additional file 1: Fig. S4B).

The strains which were grown on cellobiose showed also higher PHB yield per consumed sugar, in comparison to strains which were grown on glucose. During the first 72 h, the PHB per consumed sugar was low in both glucose cultivations: 9.3 mg PHB g^−1^ glucose with free glucose and 12.9 mg PHB g^−1^ glucose with EnPump 200 system (Additional file 1: Fig. S4C). For comparison, by 72 h, the cellobiose-consuming strains PHB_cbp and PHB_GH1-1 had 7.0 and 3.7-fold higher PHB yields per c mol of consumed sugar (p < 0.0005), respectively, in comparison to strain PHB_glu.

### Bioreactor cultivations

**Fig. 5:**
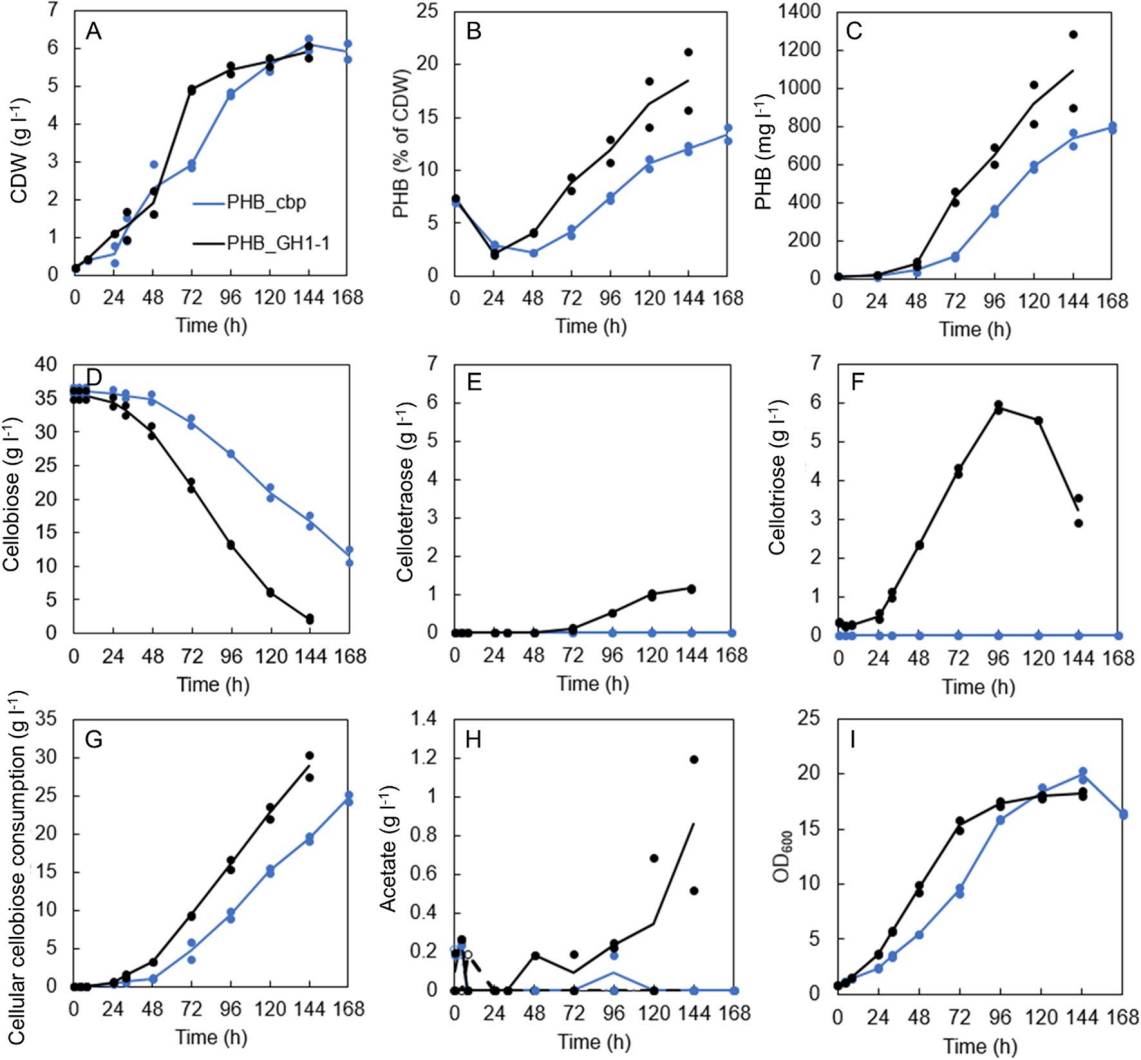
Results of batch cultivations of cellobiose utilizing PHB producing strains in bioreactors. Lines present averages of two replicates. Individual data points are marked with circles to visualize the range of measured data

To gain insight in the scale-up possibility of PHB production from cellobiose, batch cultivations were conducted in 1 L benchtop bioreactors on 35 g l^−1^ cellobiose. The strain expressing *GH1-1* gene (PHB_GH1-1) consumed nearly all cellobiose during the 144 h- cultivation, having only 2.0 g l^−1^ cellobiose left, while the strain expressing *cbp* gene(PHB_cbp) consumed around half of the cellobiose, with 16.6 g l^−1^ left at 144 h (Fig. 5D). We also noticed a formation of extracellular cellotriose and cellotetraose by the strains expressing *GH1-1* gene (Fig. 5E, F). None of the analyzed strains produced ethanol in the experiment. Acetate production of the PHB_GH1-1 strain (around 1 g l^−1^ by 144 h) is presented in Fig. 5H.

Both strains had two distinct growth phases during the cultivation. They started to grow fast around 24 h and continued growing fast for next 48 (PHB_GH1-1) or 72 h (PHB_cbp). The GH1-1_control and cbp_control strains grew similarly to their corresponding PHB strains (Additional file 1: Fig. S5A, B). The PHB_GH1-1 strain showed steady increase in the intracellular PHB already from 24 h onwards, whereas the PHB_cbp strain showed an increase in PHB levels only from 48 h onwards (Fig. 5B). Maximum growth rates, specific growth rates, PHB productivities, and specific PHB productivities are presented in Table 2. Both strains consumed similar amount of cellobiose during their fast growth phases, on average 9.4 g l^−1^, but used it differently. The PHB_cbp strain produced on average 8% more biomass (p = 0.03), but 19% less PHB (p = 0.11) per consumed cellobiose in comparison to the PHB_GH1-1 strain (Table 2). However, this 19% difference in PHB yield per consumed sugar is not significant due to high variation in PHB accumulation in PHB_GH1-1 replicates (Fig. 5B). This applies also to observed 40% higher maximum PHB productivity and 80% higher maximum specific PHB productivity of the PHB_GH1-1 strain, in comparison to PHB_cbp strain (Table 2).

**Table 2:** Bioreactor results during the fast growth phase for each replicate

Strain	Fast growth phase	Maximum growth rate (mg CDW l^−1^ h^−1^)	Maximum specific growth rate (h^−1^)	Biomass yield per cellobiose (mg g^−1^)	Max PHB productivity (mg PHB l^−1^ h^−1^)	Max specific PHB productivity (mg PHB g CDW^−1^ h^−1^)	PHB yield on cellobiose (mg g^−1^)	Max PHB accumulation per biomass (% of CDW)
PHB_cbp	24–96 h	0.07 (at 77.5 h)	0.061 (at 30.1 h)	470	11.5 (at 90.3 h)	2.81 (at 82.5 h)	37.9	7.7 (at 96 h)
PHB_cbp	24–96 h	0.07 (at 84 h)	0.062 (at 28.1 h)	466	10.5 (at 96 h)	2.39 (at 87.1 h)	37.4	7.2 (at 96 h)
PHB_GH1-1	24–72 h	0.092 (at 58.5 h)	0.032 (at 58.1 h)	440	13.4 (at 62.6 h)	3.84 (at 54.9 h)	43.1	8.1 (at 72 h)
PHB_GH1-1	24–72 h	0.106 (at 59.2 h)	0.047 (at 56.9 h)	428	17 (at 62.5 h)	5.6 (at 53.9 h)	49.4	9.4 (at 72 h)

*CDW* cell dry weight, *PHB* polyhydroxybutyrate

PHB was extracted from bioreactor samples in the end of each experiment, at 144 h from strain PHB_GH1-1 and at 168 h from strain PHB_cbp. Polymer properties were analyzed from the extracted polymer with SEC. The PHB_cbp strain produced PHB polymer with on average 10% higher number average molecular weight (Mn, 229 kDa) and 12% higher weight average molecular weight (Mw, 504 kDa) than the PHB_GH1-1 strain (Table 3). However, these differences were not significant due to high variation between replicates, 24-h longer cultivation time for the PHB_cbp strain, and robust nature of the SEC analysis. Corresponding chromatograms are presented in Additional file 1: Fig. S6.

**Table 3:** Molecular weights of the extracted PHB polymers

Strain	Mn (kDa)	Mw (kDa)	*Đ*
PHB_cbp	242	526	2.2
PHB_cbp	216	482	2.2
PHB_GH1-1	196	444	2.3
PHB_GH1-1	218	460	2.1
PHB standard^a^	258	563	2.18

*Mn* number average molecular weight, *Mw* weight average molecular weight, *Đ* dispersity

^a^Data published by Ylinen et al. [9]

## Discussion

In this study, we demonstrated PHB production from cellobiose by *S. cerevisiae*. We showed that *S. cerevisiae* can synthesize PHB when PHB pathway, cellodextrin transporter Cdt-1 and either of the two enzymes metabolizing cellobiose, Cbp or Gh1-1, are introduced. The PHB titer and accumulation of biomass were highly dependent on the cellobiose utilization rate. The strain with β-glucosidase, PHB_GH1-1, consumed cellobiose faster than the strain with cellobiose phosphorylase, PHB_cbp, both in shake flasks and in bioreactors. The higher sugar consumption resulted in faster growth and PHB accumulation. However, it did not improve biomass formation per consumed sugar. For example, during the fast growth phase in bioreactors, the PHB_GH1-1 had 7% lower biomass yield per consumed cellobiose, in comparison to strain PHB_cbp strain. In addition, the PHB_GH1-1 strain converted around 12% of cellobiose to cellotriose and cellotetraose. This conversion has been reported also earlier and explained by the transglycosylation activities of the Gh1-1 enzyme followed by efflux of the cellodextrins [15, 35]. Each transglycosylation reaction results in the loss of one glucose molecule that would otherwise enter the glycolytic pathway. Both transport and utilization are less efficient for longer cellodextrins than for cellobiose, therefore, this decreases the overall cellobiose utilization efficiency of the strain expressing *GH1-1*. Removing or decreasing the transglycosylation activity could be an interesting approach for future enzyme engineering. For a fair comparison of the two enzymes used, we calculated all yields on the actual consumed cellobiose, subtracting the carbon that was converted to longer cellodextrins. The faster growth of *S. cerevisiae* strains expressing *GH1-1* gene, in comparison to strains expressing cellobiose phosphorylase gene, has been discussed in two cellodextrin transport studies [15, 16]. The difference is attributed to the Cbp enzyme having an over two-fold higher Michaelis constant k_m_ for cellobiose than Gh1-1 and the phosphorylation reaction being thermodynamically less favorable compared to hydrolysis. Ha et al. proposed that in strain expressing *cbp* gene, a higher intracellular cellobiose concentration is required to drive the cellobiose metabolism at the same rate as in the strain expressing *GH1-1* gene [15].

We observed higher cellobiose consumption in the bioreactors than in shake flasks: 27–63% of available cellobiose was consumed in bioreactors by 96 h, while in shake flasks the same strains consumed only 14–18% of available cellobiose in 96 h. The better cellobiose consumption in the bioreactor is probably related to improved growth conditions, including pH control (pH 6). In *S. cerevisiae* the Cdt-1 transporter becomes less efficient when the pH drops, with very little transport below pH 4 [23]. Thus, in the unbuffered flask experiments the cellobiose transport rate would have decreased over time. Recently described mutated variants of the *N. crassa* Cdt-2 cellobiose transporter have improved cellobiose transport rates and acid tolerance, and could potentially overcome the problem with the low pH [20].

In bioreactor cultivations, the PHB_GH1-1 strains accumulated on average 18% PHB of CDW on cellobiose. This is a higher PHB content per CDW than previously reported for *S. cerevisiae* on glucose (9–11% of CDW) [9, 25] or on xylose (15–16% of CDW) [2, 36]. In addition, in this study, the PHB accumulation in bioreactors on cellobiose was higher than in shake flasks on cellobiose (10.5% of CDW) or in shake flasks on glucose (6.1% of CDW). Earlier studies highlight especially the importance of sufficient cofactor and precursor availability for the PHB production [25, 37–41]. The early PHB accumulation levels of 0.6–7.5% PHB of CDW, have been increased e.g., by enhancing availability of precursor acetyl-CoA and its conversion to 3-hydroxybutyryl-CoA. We did not observe ethanol formation in our bioreactor cultures with any of the cellobiose consuming strains confirming that their metabolic states were fully respirative. In a fully respirative metabolic state *S. cerevisiae* metabolizes larger fraction of sugars via pentose phosphate pathway, generating NADPH, in comparison to fermentative metabolic states [42, 43]. Respirative metabolism in cellobiose utilizing strains might thus promote synthesis of NADPH demanding products such as PHB.

It is interesting that the growth on EnPump 200 media resembled the growth pattern on cellobiose, but the PHB yield per consumed sugar was lower on EnPump 200 media than on cellobiose. This indicates that cellobiose utilization benefits the PHB production with respect to glucose.

Previous studies have shown possible energetic benefits using a cellobiose phosphorylase over a β-glucosidase under anaerobic or acid stress conditions, resulting in increased biomass and ethanol yields [15, 16]. Here, in the shake flask cultivations, we observed slower growth, but higher PHB accumulation (% of CDW) in the strain expressing *cbp* gene instead of *GH1-1* gene. However, in the bioreactor cultivations, the strains expressing *GH1-1* gene showed higher PHB accumulation (% of CDW), productivity (mg PHB l^−1^ h^−1^), and yield per consumed sugar than the cbp strain. The biomass yield per cellobiose was nevertheless slightly higher for the strain expressing *cbp* gene. These observations suggest that benefits are strain and cultivation condition specific, and it could be possible that using different cellobiose transporters and anaerobic cultivation conditions would benefit the strains expressing *cbp* gene.

Both strains grown on cellobiose produced PHB with Mw of approximately 500 kDa when cells were grown in bioreactor for 144 to 168 h. These high molecular weights indicate that polymer properties are suitable for applications requiring high crystallinity and thermoplastic properties [44]. The measured molecular weights were approximately two-fold higher than in study where PHB was extracted from yeast *Yarrowia lipolytica* (200 kDa Mw) [33].

## Future

Cellobiose strains were able to convert the available sugar more efficiently to PHB, in comparison to glucose strains. In addition, cellobiose strains produced high molecular weight PHB polymer suitable for many applications. These results demonstrate that the presented cellobiose system is an interesting platform for production of PHAs and other acetyl-CoA based products. Especially PHAs are excellent alternatives for petrochemical plastics and their sustainable production will become highly important in the near future. PHA production has been traditionally studied growing cells on glucose originating from edible crops. However, the use of other carbon sources, such as lignocellulose-derived cellobiose and xylose [36], could increase both sustainability and profitability of the bioproduction. In addition, the co-fermentation of both of these sugars simultaneous could benefit the PHB production in multiple ways, as shown for ethanol production in literature [45]. Co-fermentation could provide the cells more carbon faster and enable higher utilization rate of the original biomass, without risk of high glucose repression. Cellobiose utilization could be enhanced also by increasing cellobiose transporter activities at low pH. This would improve the use of cellobiose in naturally acid tolerant *S. cerevisiae* strains in industrial fermentation conditions. PHB production could be also further improved by additional optimization of the precursor supply as discussed in literature. For example, improved expression of engineered acetyl-CoA synthetase gene (*acsL641P*) and acetaldehyde dehydrogenase gene (*ALD6*) have shown positive effects on PHB production in *S. cerevisiae* [46]. In addition, PHA production in yeast could potentially benefit from the extensive engineering efforts in acetyl-CoA and lipid production, which have resulted already in accumulation of high concentrations of triacylglycerols, 65% and 80% of CDW, in yeasts *S. cerevisiae* and *Y. lipolytica*, respectively [6, 47, 48].

## Conclusions

In this study, we produced PHB from cellobiose in *S. cerevisiae* strains expressing a cellobiose transporter gene together with either a β-glucosidase or a cellobiose phosphorylase gene. These strains showed intracellular PHB accumulation that exceeded that of a strain producing PHB from glucose.

## Supplementary Information

**Additional file 1: Figure S1.** Linear regression function built with cell dry weight (CDW) (mg l^−1^) and OD_600_ data from bioreactors (CDW = 0.3181 * OD_600_—0.0463). **Figure S2.** Polynomial function for estimation on cellobiose concentration in PHB_GH1-1 bioreactors. **Table S1.** Results from the first flask experiment (Fig. 2). **Figure S3.** Glucose released to culture media from EnPump 200 reagent in the 96-h flask experiment (Fig. 4). **Figure S4.** A: The estimated cell dry weight (CDW) values in the 96-h flask experiment. B: Estimated PHB titer as g l^−1^, C: Estimated PHB yield as g g^−1^ sugar. **Table S2.** Bioreactor results during the slow growth phase for each replicate. **Figure S5.** Results from bioreactor cultivation including controls strains cbp_control and GH1-1_control (dashed lines). The PHB producing strains were analyzed in two replicates and their control strains in one replicate. The PHB strains (continuous lines) are shown here for clarity, they are also presented in Fig. 5. **Figure S6.** The SEC chromatograms of the PHB polymer extracted from strain PHB_cbp and PHB_GH1-1. **Figure S7.** Cell growth and cellobiose consumption of strain PHB_glu grown on synthetic complete media supplemented with cellobiose in shake flasks during the 72-h cultivation.

## Data Availability

All relevant data generated or analyzed during this study are included in this published article (and its additional files).

## Abbreviations

3HB: 3-Hydroxybutyric acid
3HB-CoA: 3-Hydroxybutyryl-CoA
Cbp: Cellobiose phosphorylase
Cdt-1: Cellodextrin transporter
CDW: Cell dry weight
CoA: Coenzyme A
GC–MS: Gas chromatography mass spectrometry
Gh1-1: β-Glucosidase
Mn: Number average molecular weight
Mw: Weight average molecular weight
PHA: Poly(hydroxyalkanoate)
PhaA: Acetyltransferase
PHB: Polyhydroxybutyrate
PhaB1: Acetoacetyl-CoA reductase
PhaC1: PHA synthase
SEC: Size exclusion chromatography
