# Supplementary material for: PHB production from cellobiose with Saccharomyces cerevisiae
Source: Microb Cell Fact. 2022 Jun 21;21:124. doi: 10.1186/s12934-022-01845-x (PMC9210708; doi:10.1186/s12934-022-01845-x)
Supplement: Supplementary file 1 — Additional file 1: Figure S1. Linear regression function built with cell dry weight (CDW) (mg l−1) and OD600 data from bioreactors (CDW = 0.3181 * OD600—0.0463). Figure S2. Polynomial function for estimation on cellobiose concentration in PHB_GH1-1 bioreactors. Table S1. Results from the first flask experiment (Fig. 2). Figure S3. Glucose released to culture media from EnPump 200 reagent in the 96-h flask experiment (Fig. 4). Figure S4. A: The estimated cell dry weight (CDW) values in the 96-h flask experiment. B: Estimated PHB titer as g l−1, C: Estimated PHB yield as g g−1 sugar. Table S2. Bioreactor results during the slow growth phase for each replicate. Figure S5. Results from bioreactor cultivation including controls strains cbp_control and GH1-1_control (dashed lines). The PHB producing strains were analyzed in two replicates and their control strains in one replicate. The PHB strains (continuous lines) are shown here for clarity, they are also presented in Fig. 5. Figure S6. The SEC chromatograms of the PHB polymer extracted from strain PHB_cbp and PHB_GH1-1. Figure S7. Cell growth and cellobiose consumption of strain PHB_glu grown on synthetic complete media supplemented with cellobiose in shake flasks during the 72-h cultivation. [file 12934_2022_1845_MOESM1_ESM.docx]

**Additional file 1**

Title: “PHB production from cellobiose with *Saccharomyces cerevisiae*”

Anna Ylinen^1^, corresponding author

Jorg C. de Ruijter^1^

Paula Jouhten^1,2^

Merja Penttilä^1, 3^

^1^ VTT Technical Research Centre of Finland Ltd. P.O. Box 1000, FI-02044 VTT, Finland

^2^ Current address: Department of Bioproducts and Biosystems, School of Chemical Engineering, Aalto University, P.O. Box 11000, FI-00076 AALTO, Finland

^3^ Department of Bioproducts and Biosystems, School of Chemical Engineering, Aalto University, P.O. Box 11000, FI-00076 AALTO, Finland


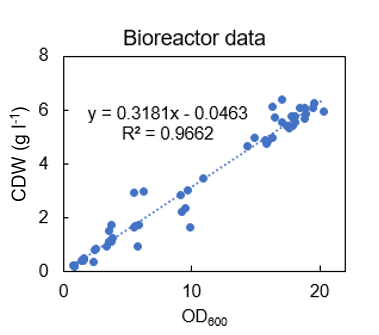


Figure S1. Linear regression function built with cell dry weight (CDW) (mg l^-1^) and OD_600_ data from bioreactors (CDW = 0.3181 * OD_600_ - 0.0463). Data points represent measured values from PHB_cbp, cbp_control, PHB_GH1-1, and GH1-1_control strains.

Figure S2. Polynomial function for estimation on cellobiose concentration in PHB_GH1-1 bioreactors.


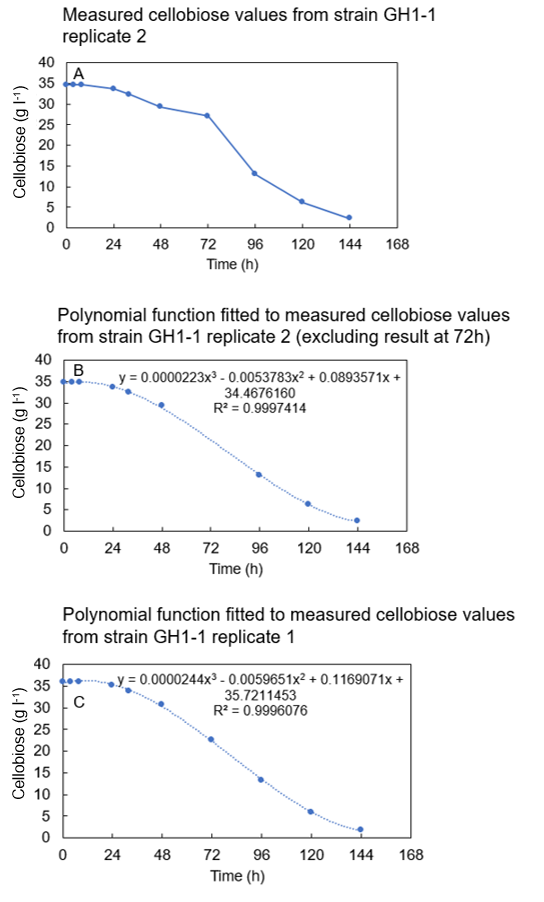


Table S1. Results from the first flask experiment (Fig 2). The PHB producing strains and control strains were grown in flasks for 72 hours with glucose or cellobiose. The CDW values were estimated based on measured OD_600_ values from the flasks and linear regression function (CDW = 0.3181 * OD_600_ - 0.0463) of the OD_600_ and CDW values from the bioreactors (Figure S2).

| **Strain** | **Carbon source** | **PHB (mg l^-1^)** | **PHB (mg g^-1^ cellobiose or glucose)** | **CDW (mg g^-1^ cellobiose or glucose)** | **PHB (% of CDW)** | **CDW (g l^-1^)** | **Cellobiose or glucose consumption (g l^-1^)** |
| --- | --- | --- | --- | --- | --- | --- | --- |
| PHB_glu | Glucose | 108.4 ± 18 | 5.3 ± 0.9 | 141 ± 0 | 3.8 ± 0.6 | 2.9 ± 0.01 | 20.3 ± 0.1 |
| PHB_cbp | Cellobiose | 25.5 ± 2.8 | 17.7 ± 5.6 | 495 ± 0.11 | 3.7 ± 0.5 | 0.7 ± 0.07 | 1.4 ± 0.2 |
| PHB_GH1-1 | Cellobiose | 17.6 ± 0.1 | 7.8 ± 0.5 * | 334 ± 0.08 * | 2.4 ± 0.1 | 0.7 ± 0.02 | 2.2 ± 0.1 |
| cbp_control | Cellobiose | 0.0 | 0.0 | 469 ± 0.03 | 0.0 | 0.8 ± 0.08 | 1.8 ± 0.1 |
| GH1-1_control | Cellobiose | 0.0 | 0.0 | 273 ± 0.08 * | 0.0 | 0.7 ± 0.04 | 2.6 ± 0.7 |
| CEN.PK111-9A | Glucose | 0.0 | 0.0 | 143 ± 0 | 0.0 | 2.8 ± 0.13 | 19.5 ± 0.7 |

*The possible cellobiose conversion cellotriose or cellotetraose production was not measured nor considered in the calculation. Later bioreactor experiment shows that by 72 hours, the cells expressing *GH1-1* gene could have converted around 30% of the consumed cellobiose to longer cellodextrins. If such conversion occurred in this experiment, the PHB and biomass yield per consumed cellobiose would be higher than presented here.


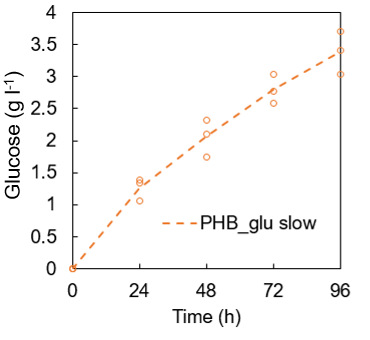


Figure S3. Glucose released to culture media from EnPump 200 reagent in the 96-hour flask experiment (Figure 4). Strain: PHB_glu.


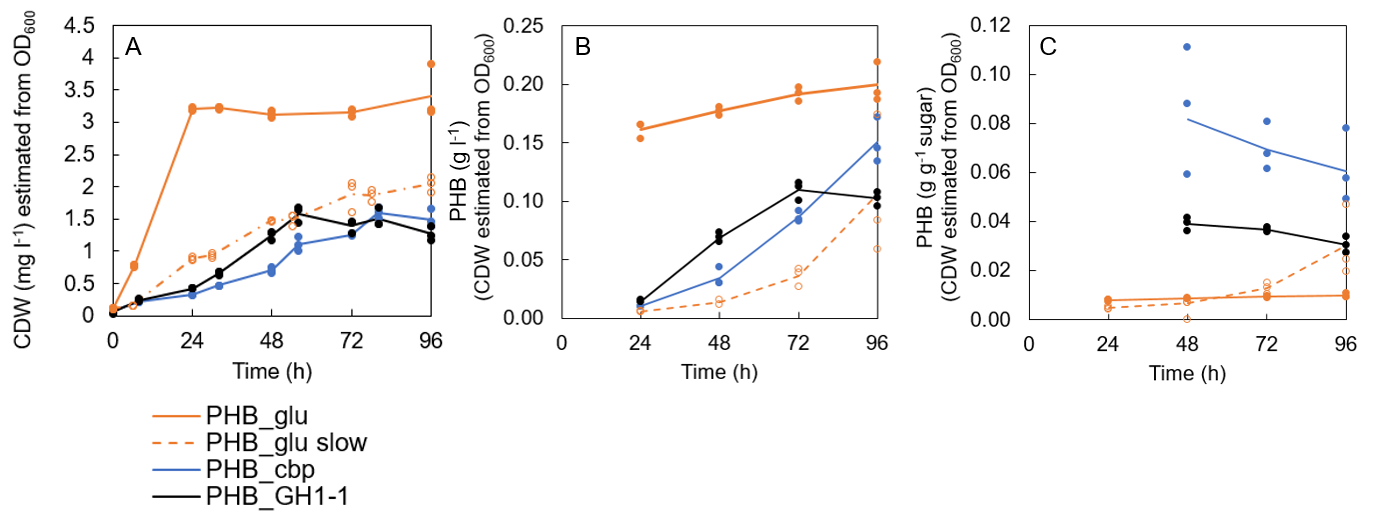
 Figure S4. A: The estimated cell dry weight (CDW) values in the 96-hour flask experiment. The CDW values were predicted based on measured OD_600_ values from the flasks (Figure 4) and linear regression model of the OD_600_ and CDW values from the bioreactors (Figure S1). B: Estimated PHB titer as g l^-1^, C: Estimated PHB yield as g g^-1^ sugar. Strains PHB_cbp and PHB_GH1-1 were grown with 20 g l^-1^ cellobiose and strain PHB_glu with either 20 g l^-1^ glucose (PHB_glu) or with EnPump 200 slow glucose release media (PHB_glu slow). Lines represent averages of two or three biological replicates. Individual data points are presented with circles.

Table S2. Bioreactor results during the slow growth phase for each replicate. Abbreviations: CDW: cell dry weight; PHB: polyhydroxybutyrate.

| **Strain** | **Slow growth phase** | **Biomass yield per cellobiose (mg g^-1^)** | **Max PHB accumulation per biomass (% of CDW)** | **PHB yield on cellobiose (mg g^-1^)** |
| --- | --- | --- | --- | --- |
| PHB_cbp | 96h-144h | 156 | 12.3 | 44 |
| PHB_cbp | 96h-144h | 110 | 11.8 | 33 |
| PHB_GH1-1 | 72h-144h | 38 | 15.7 | 43 |
| PHB_GH1-1 | 72h-144h | 66 | 21.3 | 71 |


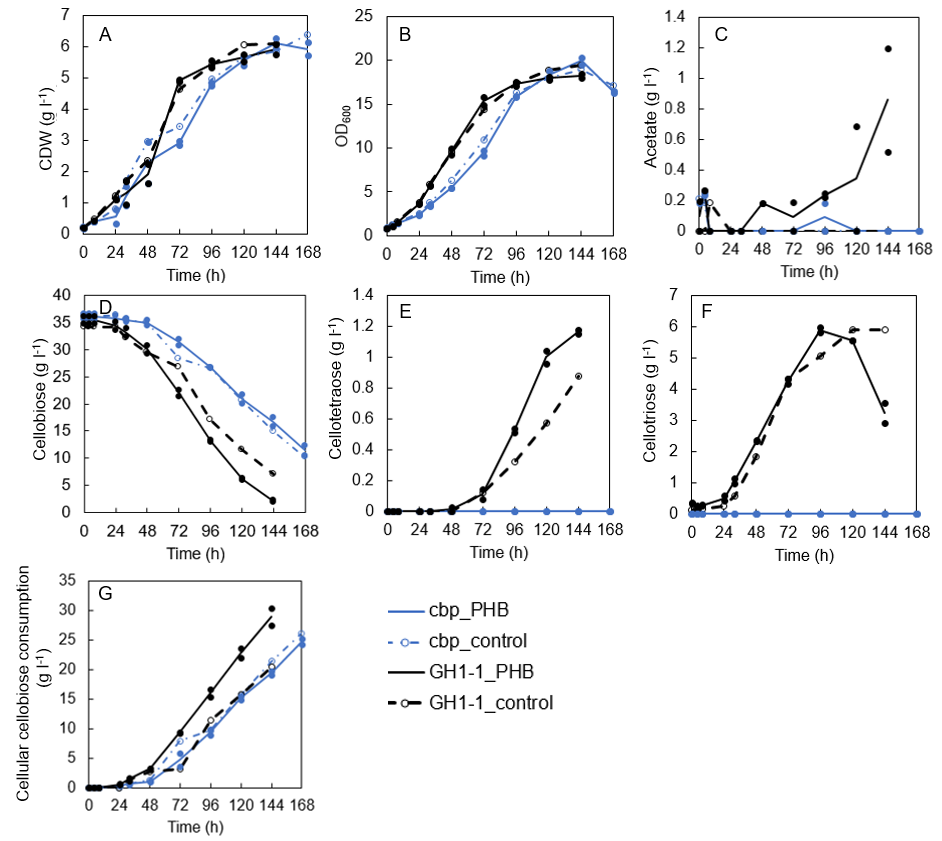


**Figure S5.** Results from bioreactor cultivation including controls strains cbp_control and GH1-1_control (dashed lines). The PHB producing strains were analyzed in two replicates and their control strains in one replicate. The PHB strains (continuous lines) are shown here for clarity, they are also presented in Figure 5.


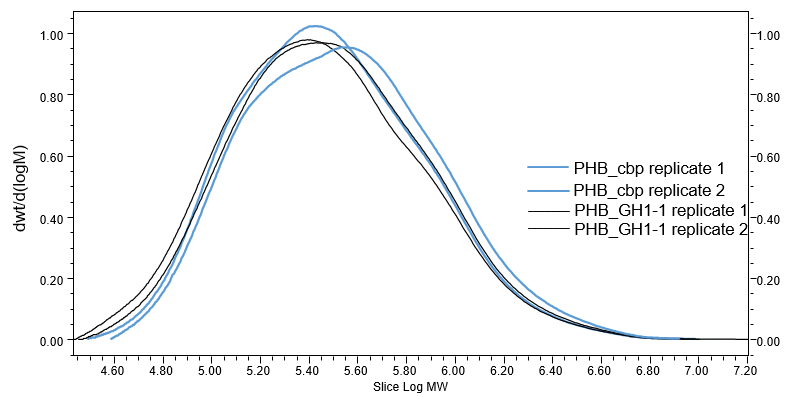


Figure S6. The SEC chromatograms of the PHB polymer extracted from strain PHB_cbp and PHB_GH1-1.


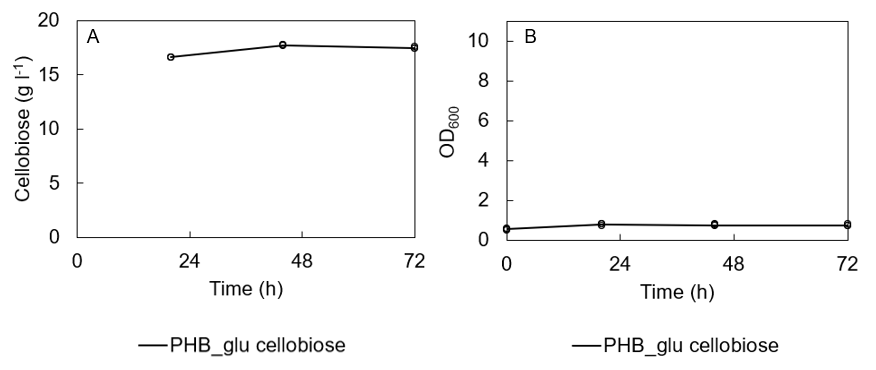


Figure S7. Cell growth and cellobiose consumption of strain PHB_glu grown on synthetic complete media supplemented with cellobiose in shake flasks during the 72-hour cultivation. Individual data points are presented with circles to visualize the range of measured data.
